# Assessment of photobiomodulation in response to the microcirculation in arteriovenous fistula for hemodialysis patient

**DOI:** 10.2478/abm-2025-0005

**Published:** 2025-02-28

**Authors:** Yi-Ping Chang, Chuan-Tsung Su, Ya-Hui Hsieh, Fan-Chieh Meng, Jih-Huah Wu

**Affiliations:** Department of Nephrology, Taoyuan Branch of Taipei Veterans General Hospital, Taoyuan 333, Taiwan; Department of Healthcare Information and Management, Ming Chuan University, Taoyuan 333, Taiwan; Division of Cardiovascular Surgery, Ministry of Health and Welfare Taipei Hospital, New Taipei City 242033, Taiwan; Department of Biomedical Engineering, Ming Chuan University, Taoyuan 333, Taiwan

**Keywords:** harteriovenous fistula, cumulative effect, hemodialysis, microcirculation, photobiomodulation

## Abstract

**Background:**

Management of blood flow in arteriovenous fistula (AVF) is a critical clinical issue for hemodialysis (HD) patients.

**Objectives:**

To determine the effect of microcirculation of AVF in HD patients with photobiomodulation (PBM).

**Methods:**

Twenty HD patients were enrolled in this study. PBM was used to radiate the palm of HD patients at a total dose of 126 J, and the microcirculatory analysis in AVF was investigated.

**Results:**

Among the patients <65 years old, there is an increase of 2.31% and 1.37% in the average velocity and flux in AVF, respectively. This increase is higher than those observed in patients aged >65 years old. Additionally, the cumulative effect of the 830 nm laser was observed for at least 10 min, resulting in continuous increases of 3.16% in velocity and 1.59% in flux for HD patients <65 years old. On the contrary, the average velocity and flux in AVF increased in patients who had undergone HD for <6 years.

**Conclusions:**

In this study, the age and the duration of HD treatment are the two factors that influence microcirculation in HD patients with PBM. The results suggest that PBM could be used to improve the average velocity and flux in AVF, particularly for younger patients with shorter HD treatment durations.

The end-stage renal disease (ESRD) is an advanced and permanent stage of chronic kidney disease (CKD), characterized by a significant decline in kidney function to the extent that the kidneys are incapable of performing essential bodily functions. Either renal replacement therapy (RRT) or kidney transplantation is necessary for the survival of ESRD patients. The global prevalence of CKD exceeds 13% [1], with over 2.5 million individuals' dependents on RRT [2]. The modality of RRT can be divided into hemodialysis (HD) and peritoneal dialysis. HD is the predominant dialysis modality worldwide, with over 80% of chronic dialysis patients underwent HD in-center [3]. Typically, arteriovenous fistulas (AVFs), artificial arteriovenous grafts (AVGs), and central venous catheters (CVCs) are the 3 types of vascular access (VA) used in HD patients. In general, AVF or AVG is commonly used as maintenance HD therapy. Clinical guidelines encourage the use of AVF than AVG because of its higher patency rate and lower complication rate once established. However, the cost of AVF or AVG is a significant burden on Medicare budgets and patients [4], and it is often limited by non-use, loss of patency, and abandonment rates [5]. VA dysfunction is a major cause of morbidity, mortality, and high healthcare costs in HD patients [6, 7]. Feldman et al. [8] indicated that hospitalization costs associated with VA complications in HD patients may exceed $1 billion annually in the United States. When comparing the cost of VA per patient-year at risk, AVGs are the highest, followed by CVCs, and AVFs are the lowest [9]. HD therapy represents a growing challenge in clinical practice as well as a financial burden on healthcare systems worldwide. Therefore, it is critical to select an effective method to improve the functional status of VA, especially AVF in ESRD patients.

The most common complications of VA include lymphedema, infection, aneurysm, stenosis, congestive heart failure, steal syndrome, ischemic neuropathy, and thrombosis [10]. Stenosis is a major cause of AVF dysfunction [11, 12]. Thrombophilia has been suggested as a possible mechanism leading to dialysis access thrombosis [13]. Thrombosis is a common complication of AVF, whether early or late, which can eventually lead to AVF function loss. The patients who underwent VA surveillance had a median thrombosis rate of 0.33 events per 1,000 patient days (7 groups; 721 fistulas) [14]. Thrombosis may cause about 80%–85% of VA failures in HD patients, and >80% of these are caused by underlying VA stenosis [15]. Thus, stenosis and thrombosis are two major factors that could cause lower blood flow in VA. The critical pathogenesis for stenosis and frequent thrombosis is adequate vascular remodeling and neointimal hyperplasia [16].

Maintaining the patency and adequate blood flow are crucial for successful HD therapy. There are several methods to be utilized except basic hand exercises, such as medications [16,17,18], percutaneous intervention [19], radiological intervention [20], and far infrared (FIR) therapy [21,22,23,24,25]. An alternative and complementary method is photobiomodulation (PBM), which may have an adjunctive effect for improving blood flow in dialysis VA. The therapeutic applications of PBM have remarkably expanded in the past decades, such as cell proliferation rate [26, 27], tissue regeneration [28], clinical pain management [29,30,31,32], and microcirculation in animal models [33, 34]. Studies have demonstrated that PBM can improve collateral circulation and microcirculation in rabbits [33] and increase arteriolar blood flow in rat mesenteric microcirculation [34]. Furthermore, PBM is considered a safe modality with no cytotoxic or genotoxic potential, as highlighted by Logan et al. [35]. Recently, near-infrared (NIR) light (750–1,100 nm) radiated on blood is effective in reducing oxidative stress during HD, and it could be considered as a concurrent pretreatment strategy for HD treatment [36]. Functional capacity and muscle strength of chronic kidney failure patients can be improved after 8 weeks of PBM therapy [37]. PBM effectively alleviates mitochondrial dysfunction, reactive oxidative stress, inflammation, and gut microbiota dysbiosis, all of which are inherent in CKD [38]. In addition, PBM has been shown to be effective in relieving itching pain [39], improving hyposalivation and urea levels in saliva [40], and alleviating restless legs syndrome [41] in patients undergoing HD. Recently, PBM has been shown to have a vasodilation effect in several animal studies [42,43,44]. Keszier et al. [42] established a mouse model, and their results showed that PBM can regulate blood vessel dilation by releasing a vasoactive nitric oxide precursor species. PBM is capable of inducing a long-lasting hypotensive effect in hypertensive rats, and this effect may be attributed to vasodilation via a nitric oxide-dependent mechanism [43]. Also, the vasodilation effect inducing hypotension in rats by PBM was observed by Buzinari et al. [44]. Therefore, the beneficial effects of PBM could be used for clinical applications in HD patients.

Nawashiro et al. [45] found that a patient in a persistent vegetative state, who received 850 nm light-emitting diode (LED) on the left and right forehead areas, exhibited a 20% increase in cerebral blood flow (CBF) in the left anterior frontal lobe. In addition, the mean CBF responses dependent on the light dose was investigated by Rungta et al. [46]. Recently, using a mixture of 625 nm, 660 nm, and 850 nm LED devices to radiate on chronic wounds in diabetic and non-diabetic patients were investigated [47]. The results showed that the mean blood flow could be improved in the treated group of both diabetic and non-diabetic patients by using laser Doppler flowmetry (LDF) measurement. In a recent study, 660 nm LED array system was used to radiate on the palm of the hand, revealing an approximately 1.7-fold increase in nailfold microcirculation [48]. In other study, 830 nm LED induced a 27% increase in microcirculatory flow, which further increased to 54% during the 20 min follow-up period, as measured by LDF [49]. Additionally, skin microcirculation was assessed at the upper limb contralateral to the AVF using LDF to determine any changes in uremic patients and evaluate the effects of HD session [50]. Besides, the periflux blood flow decreasing velocity of patients who undergwent HD can be assessed by LDF. Furthermore, the periflux blood flow decreasing velocity of patients undergoing HD can be assessed using LDF [51]. On the contrary, FIR therapy is another recommended and convenient therapeutic method that has been applied to HD patients [21,22,23,24,25]. The wavelength for FIR ranges from 50 μm to 1,000 μm, exhibiting a high absorption coefficient for water with a value of 10^3^ cm^−1^ [52]. However, a lower absorption coefficient (10^−1^ cm^−1^) of water for NIR light can be achieved. In comparison to FIR, the water absorption coefficient of NIR is reduced by 10,000 times. This implies that NIR light has a higher penetration rate in biological tissue. In our previous study, NIR light is more suitable than the red light for deeper biotissue stimulation [53]. Therefore, the laser wavelength at 830 nm was utilized in this study.

However, the effects of activating microcirculation over the AVF with PBM have not been conclusively confirmed. Some questions remain unknown, such as whether there is a simpler way to improve the VA function in HD patients, especially in the elderly. Therefore, the microcirculatory response in AVF for HD patients with 830 nm laser was investigated in this study.

## Methods

### Laser device

**Figure 1.**
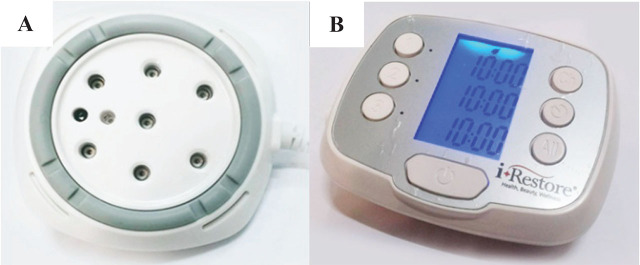
**(A)** The 830 nm laser device and **(B)** controller.

The multi-channel laser therapy system (Model: ID 310; laser array [7 laser diodes]; the aluminum gallium arsenide diode laser [Model: T8350; Pocket Laser, Opto Focus Co., Ltd.], wavelength: 830 ± 10 nm; peak emission wavelength: 830 nm; output power of each laser: 30 mW; pulse rate: 10 Hz, 50% duty cycle; 20 min; Jin-Ciang Technology Co., Ltd.), as shown in **Figure 1**, was used to radiate the palm of HD patients. The energy density of a laser is 73.16 J/cm^2^. The total treatment dose is 126 J.

### High power LDF

**Figure 2.**
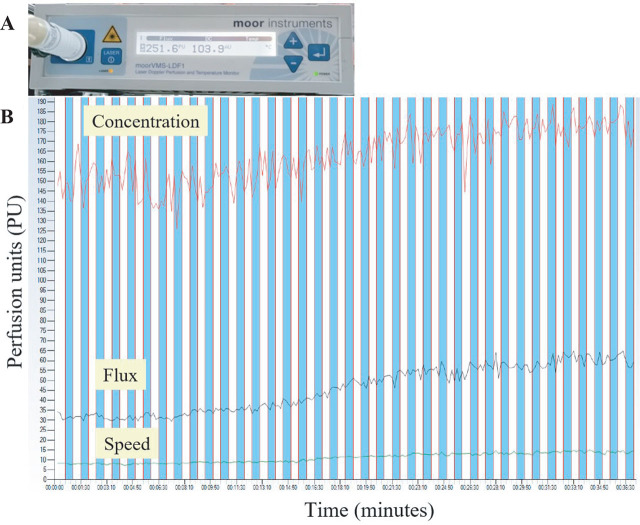
**(A)** High power LDF device and **(B)** data collection from HD patients. HD, hemodialysis; LDF, laser Doppler flowmetry.

LDF has been widely used to assess the blood flow in microcirculation. Based on the Doppler shift, the blood perfusion signal can be detected by the laser traveling through the tissue and the backscattered light from the moving red blood cells (RBCs). In this study, a high power LDF device (Model: moorVMS-LDF1 [Serial No: SN276] and moorVMS-LDF1 [Serial No: SN551], wavelength: 785 nm; maximum output power: 20 mW; continuous wave) was used to provide a quantitative measurement for assessing the microcirculation of the AVF in HD patients. The device was manufactured by Moor Instruments Ltd., UK. The high-power LDF device and data collection are shown in **Figure 2**. Microcirculatory parameters of the AVF (access blood flow, concentration, and velocity values) were analyzed and expressed as “mean ± standard deviation.” The flux and velocity of RBC in AVF could be quantitatively analyzed using perfusion units (PU) as the physical quantitative units.

### Participants

The study protocol was reviewed and approved by the Institutional Review Board of Taipei Veterans General Hospital (TPEVGHIRB: 2021-01-002B). Twenty patients (16 males and 4 females) with ESRD under maintenance HD therapy were recruited at Taipei Veterans General Hospital in Taiwan. Patients with unstable vital signs, acute infectious status, uncooperative behavior, or those using AVG or CVC for HD were excluded. The mean age of the participants was 65.5 ± 11.00 years old and the mean duration of HD was 3.99 ± 2.46 years.

### Procedure

**Figure 3.**
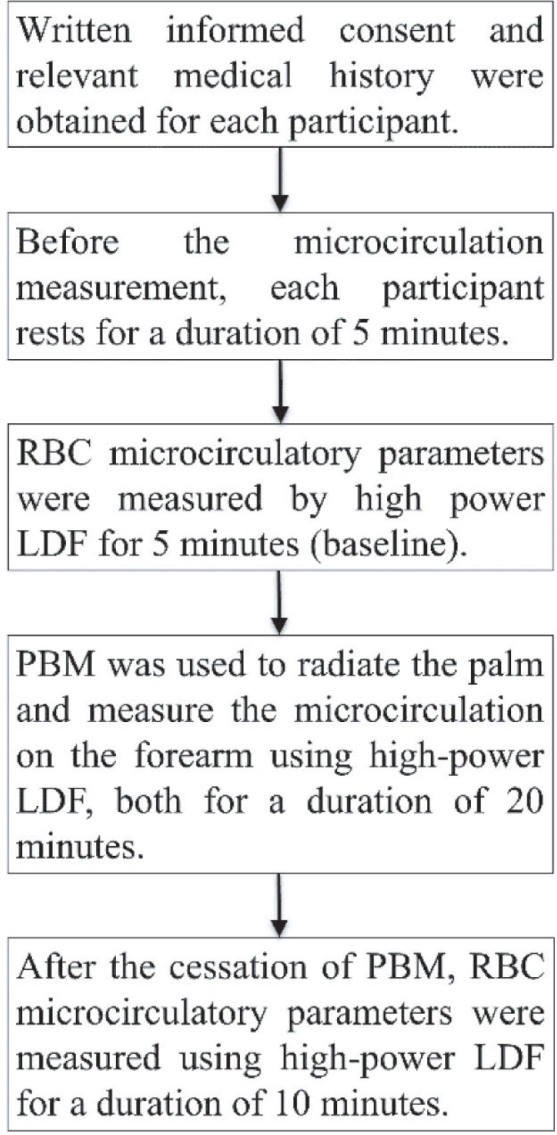
Consort flow diagram. LDF, laser Doppler flowmetry; PBM, photobiomodulation; RBC, red blood cell.

**Figure 4.**
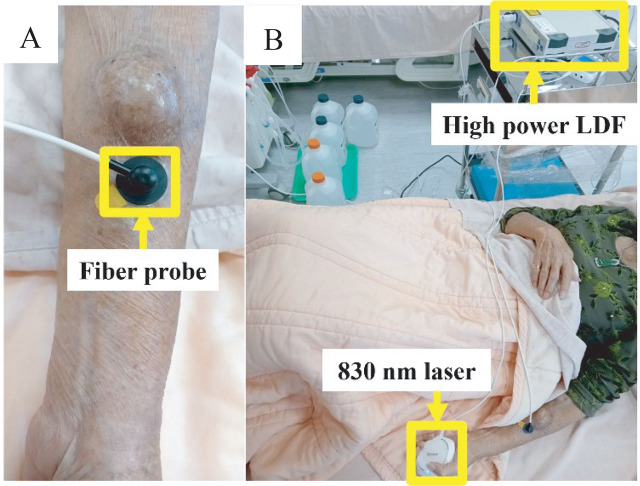
**(A)** The fiber probe of the high-power LDF was placed at the edge of the AVF. **(B)** The actual tested situation of HD patients receiving 830 nm laser on the palm. AVF, arteriovenous fistula; HD, hemodialysis; LDF, laser Doppler flowmetry.

The protocol including 3 phases in this study was followed in **Figure 3**. Each patient received one course of treatment. In the first phase, the baseline of the blood flow, concentration, and RBC velocity were measured using high-power LDF for 5 min. The fiber probe of the high-power LDF was positioned at the edge of the AVF, as illustrated in **Figure 4A**. In the second phase, 830 nm laser was used to radiate on the palm for 20 min as shown in **Figure 4B**. Concurrently, the microcirculatory parameters of the AVF were measured by high-power LDF. In the third phase, each participant underwent another 10-min session of microcirculation measurement. Finally, the flux, concentration, and velocity of RBC were compared with the baseline for each participant.

## Results

**Figure 5.**
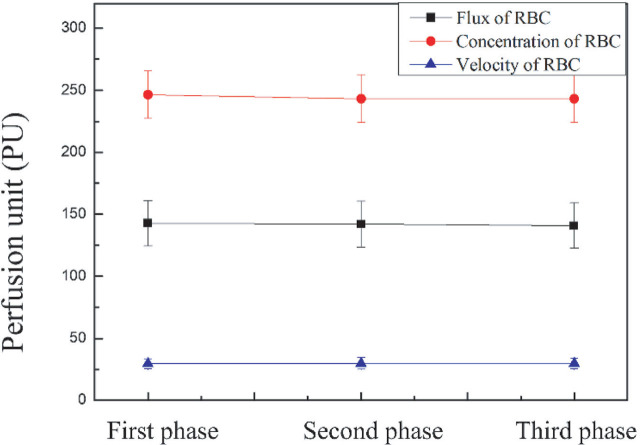
The variation of average microcirculation of AVF in HD patients with 830 nm laser (First phase: baseline; Second phase: 830 nm laser radiated on palm for 20 min; and Third phase: after the cessation of the 830 nm laser). AVF, arteriovenous fistula; HD, hemodialysis.

The variation of microcirculation in AVF of HD patients radiated by 830 nm laser was analyzed as shown in **Figure 5**. The results indicate that there is no significant difference in microcirculation in AVF for HD patients with 830 nm laser compared with the baseline.

Twenty HD patients who were undergoing different HD treatment durations were investigated, including <3 years, 3–6 years, and >6 years. For patients with HD for <3 years of treatment, the variation of the average RBC concentration was decreased by 2.18% in the second phase and continuously decreased to 3.04% in the third phase as shown in **Table 1**. The average RBC velocity shows a slight increase after the 830 nm laser cessation. For patients with HD 3–6 years of treatment, the variation of the average RBC concentration was decreased as well. The velocity and flux demonstrate 2.78% and 1.30% increased by 830 nm laser, respectively. In addition, the velocity and flux were continuously increased to 3.02% and 1.13%, respectively. For the patients with HD for >6 years of treatment, the velocity and flux were 7.25% and 6.26% decreased in the third phase, respectively.

**Table 1.** The ratio of RBC microcirculation in patients with different durations of HD period

**HD history (years)**	**Microcirculation parameters of RBC (PU)**	**Second phase (%)**	**Third phase (%)**
<3	Concentration	−2.18	−3.04
	Velocity	−0.49	+0.19
	Flux	−2.12	−2.35
3–6	Concentration	−1.03	−0.22
	Velocity	+2.78	+3.02
	Flux	+1.30	+1.13
≥6	Concentration	+0.10	+0.52
	Velocity	−0.31	−7.25
	Flux	−0.03	−6.26

HD, hemodialysis; PU, perfusion units; RBC, red blood cell.

**Figure 6.**
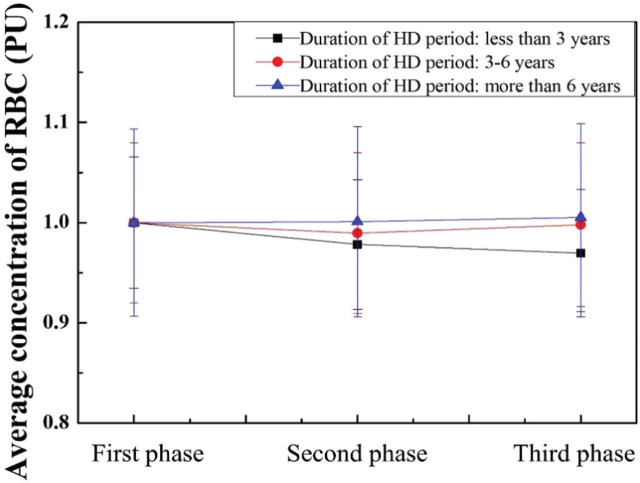
Normalized average RBC concentration in the duration of the HD period. HD, hemodialysis; PU, perfusion units; RBC, red blood cell.

The values of each phase were normalized by dividing the corresponding data in the first phase. Therefore, the average RBC concentration, velocity, and flux were normalized in AVF of HD patients with different durations of HD period, as shown in **Figure 6**. The average RBC concentration decreased when patients received an 830 nm laser with a short duration of HD.

**Figure 7.**
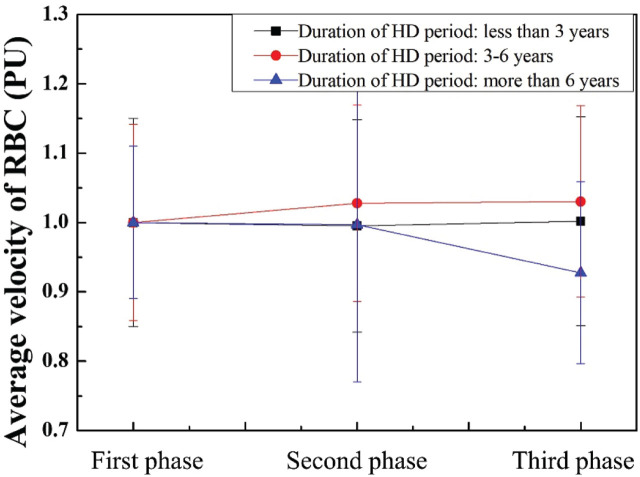
Normalized average RBC velocity in the duration of the HD period. HD, hemodialysis; PU, perfusion units; RBC, red blood cell.

The normalized average RBC velocity in the duration of the HD period is shown in **Figure 7**. For patients who had undergone HD for <6 years, the average velocity of RBC can be increased when they received 830 nm laser treatment compared with the baseline. For the patients who had undergone HD for 3–6 years, the variation of the average RBC velocity increased by 2.78%. Furthermore, the average velocity continuously increased to 3.02% when the 830 nm laser was removed. However, the average RBC velocity decreased in patients who had undergone HD for 6–9 years.

**Figure 8.**
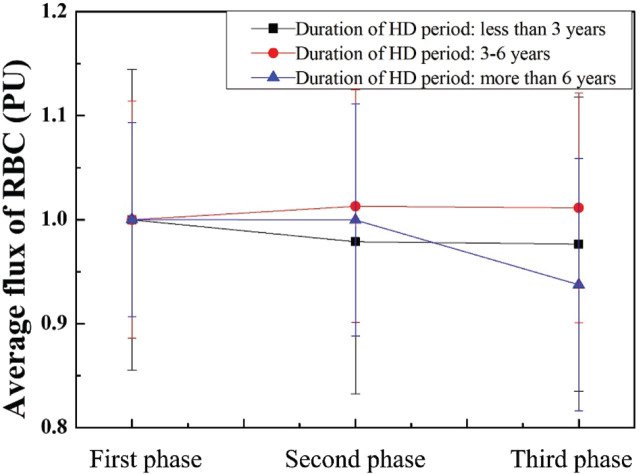
Normalized average RBC flux in the duration of the HD period. HD, hemodialysis; PU, perfusion units; RBC, red blood cell.

The normalized average RBC flux in the duration of the HD period is shown in **Figure 8**. For the patients who had undergone HD for 3–6 years, the average RBC flux increased by 1.3% and 1.13% in the second and third phases, respectively. Similarly, the average RBC flux of the patients who had undergone HD for >6 years is apparently decreased.

The average RBC velocity and flux were analyzed for HD patients of different ages as shown in **Table 2**. The results show that the average RBC velocity and flux can be increased for HD patients around the age of 60 years who received 830 nm laser radiation. For older HD patients (with an average age of approximately 70 years old), the microcirculation of RBC in AVF might not be able to increase by 830 nm laser treatment.

**Table 2.** The relationship between the age and the microcirculation in patient duration of the HD treatment

**The duration of HD period (years)**	**Number of patients with microcirculation changed in AVF (increase/decrease)**	**Age of the patient with increased microcirculation (years)**	**Age of the patient with decreased microcirculation (years)**
1	(2/3)	53.00 ± 8.49	76.00 ± 10.44
2	(1/1)	76	63
3	(1/0)	59	N/A
4	(1/2)	56	68.50 ± 6.36
5	(3/1)	66.67 ± 14.01	72
6	(1/0)	45	N/A
7	(0/3)	N/A	71.00 ± 1.00
9	(1/0)	55	N/A
Age (mean ± standard deviation)		58.67 ± 10.03	70.10 ± 4.80

AVF, arteriovenous fistula; HD, hemodialysis.

Furthermore, the ratio of RBC microcirculation in patients of different ages was analyzed, as shown in **Table 3**. For patients aged <65 years old in the second phase, the velocity and flux were 2.31% and 1.37% increased by 830 nm laser, respectively. In the third phase, the velocity and flux continuously increased to 3.16% and 1.59%, respectively. However, for patients aged >65 years old in the second phase, the velocity increased by 0.10%, whereas the flux decreased by 1.58% with an 830 nm laser. Moreover, the velocity and flux were continuously decreased by 0.90% and 3.02% in the third phase, respectively.

**Table 3.** The ratio of RBC microcirculation in patients of different ages

**Age (sample size)**	**Microcirculation parameters of RBC (PU)**	**Second phase (%)**	**Third phase (%)**
<65 (N = 9)	Concentration	−1.40	−1.48
	Velocity	+2.31	+3.16
	Flux	+1.37	+1.59
≥65 (N = 11)	Concentration	−1.34	−1.25
	Velocity	+0.10	−0.90
	Flux	−1.58	−3.02

PU, perfusion units; RBC, red blood cell.

## Discussion

Microcirculation plays a crucial role in the human body, such as providing access to oxygenated blood to tissues, maintaining global tissue blood flow, and linking local blood flow to local metabolic needs [54]. Microcirculation analysis has been studied in numerical analysis and image detection, such as image correlation method [55], capillary of binary space-time analysis [56], and RBC motion modeling [57]. AVF may have important consequences for cardiac function and limb vascularization. The determination of AVF blood flow is vital for dialysis patients. Non-invasive measurement of blood flow in HD patients has been investigated in previous studies [58, 59]. Additionally, hemodynamics model for AVF has been developed for fistula capacity, finger blood pressure, and occurrence of finger ischemia prediction [60]. Therefore, the non-invasive measurement and the prediction method are important considerations for HD patients.

Long-duration VA in chronic HD patients has been reviewed, and its side effects include thrombosis, catheter rupture, catheter malfunction, and infection [61]. Inadequate blood flow of VA in AVF could be influenced by these risk factors. The values of blood flow, concentration, and velocity of RBC in patients who had undergone different HD treatment durations were analyzed in **Figures 6–8**. In the second phase, the average concentration of RBC can be decreased by 2.18% and 1.03% in the patients who had undergone HD for <3 years and 3–6 years, respectively. In addition, the average velocity and flux of RBC can be increased by 2.78% and 1.30% in the patients who had undergone HD for 3–6 years, respectively. In the third phase, the average concentration of RBC can be decreased by 3.04% in the patients who had undergone HD for <3 years. The average velocity and flux of RBC can be increased by 3.02% and 1.13% in the patients who had undergone HD for 3–6 years, respectively. The results show that the effects persisted for at least 10 min after the cessation of laser radiation. Gavish et al. [49] demonstrated that both immediate and sustained arteriolar vasodilation can be induced by the wavelength at 830 nm.

In this study, an 830 nm laser was found to induce a 2.78% increase in the flux of RBC, which increased to 3.02% during the 10-min follow-up period. The results are similar to the previous study [49]. Based on our results, the average velocity and flow of RBC are increased in patients who had undergone HD for 3–6 years. On the contrary, the average velocity and flux of RBC were decreased in the patients who had undergone HD for >6 years. The results could be attributed to the increased concentration of RBC, which leads to low velocity of blood flux in AVF. On the contrary, the age of HD patients could affect the variation of microcirculation in AVF. **Table 2** shows that the average velocity and flux in AVF were decreased in HD patients aged approximately 70 years old who received an 830 nm laser. However, the average velocity and flux were increased in the AVF of the patients' age at approximately 60 years. The velocity and flux of HD patients aged <65 years old were higher than those aged >65 years old as shown in **Table 3**. In the second phase, 2.31% and 1.37% increase in velocity and flux in AVF were observed for HD patients with age <65 years, respectively. Moreover, the dose accumulation of 830 nm laser could be achieved in at least 10 min. The results showed that there were 3.16% and 1.59% continuous increase in velocity and flux in AVF for HD patients in the third phase, respectively. Karu et al. [62] noted the cumulative effect could be observed when the target radiated by laser. The cumulative effect was obtained when the 830 nm laser was removed in the third phase. However, lower velocity and flux were observed for the elderly (>65 years old) with an 830 nm laser. Recently, Jin [63] proposed that aging is the process of continuous impairment of microcirculation in the body. Our results suggested that age could be considered as another factor that determines the variation of microcirculation in HD patients.

The limitations of this study are as follows: First, the relatively small sample size in this study may impact the observed effects of PBM, potentially resulting in a short-term vasodilation effect in AVF. Second, this study applied a short-duration stimulation of PBM. It is imperative to explore the long-duration effects of PBM on HD patients, and the accumulation of doses should be clearly observed in the experimental data. Third, the individual variation of AVF in HD patients might affect the final results of this study. Therefore, future studies should involve a larger population and control groups. These considerations should be considered in future clinical trials. The potential effects of PBM in HD patients are necessary for further investigation.

## Conclusion

In this study, the microcirculation of the AVF in HD patients who received 830 nm laser treatment was investigated. The average velocity and flux of RBC could be increased by 830 nm laser in patients who had undergone HD for <6 years. The improvement in microcirculatory is more easily observed in younger HD patients than in elderly ones. Moreover, the continuous increase in velocity and flux in AVF for HD patients could be attributed to the cumulative effect of the 830 nm laser. This study shows that PBM might be an effective tool for the management of the blood flow of AVF in HD patients. However, further work is needed to clarify the effect of PBM in HD patients.
